# Estimates of genetic trend for single-step genomic evaluations

**DOI:** 10.1186/s12711-018-0410-1

**Published:** 2018-08-03

**Authors:** Karin Meyer, Bruce Tier, Andrew Swan

**Affiliations:** Animal Genetics and Breeding Unit, University of New England, Armidale, NSW 2351 Australia

## Abstract

**Background:**

A common measure employed to evaluate the efficacy of livestock improvement schemes is the genetic trend, which is calculated as the means of predicted breeding values for animals born in successive time periods. This implies that different cohorts refer to the same base population. For genetic evaluation schemes integrating genomic information with records for all animals, genotyped or not, this is often not the case: expected means for pedigree founders are zero whereas values for genotyped animals are expected to sum to zero at the (mean) time corresponding to the frequencies that are used to center marker allele counts when calculating genomic relationships.

**Methods:**

The paper examines estimates of genetic trends from single-step genomic evaluations. After a review of methods which propose to align pedigree-based and genomic relationship matrices, simulation is used to illustrate the effects of alignments and choice of assumed gene frequencies on trajectories of genetic trends.

**Results:**

The results show that methods available to alleviate differences between the founder populations implied by the two types of relationship matrices perform well; in particular, the meta-founder approach is advantageous. An application to data from routine genetic evaluation of Australian sheep is shown, confirming their effectiveness for practical data.

**Conclusions:**

Aligning pedigree and genomic relationship matrices for single step genetic evaluation for populations under selection is essential. Fitting meta-founders is an effective and simple method to avoid distortion of estimates of genetic trends.

## Background

Genetic evaluation based on the use of genomic information has become a routine procedure in numerous livestock improvement schemes. Many employ the so-called single-step procedure for best linear unbiased prediction (SS-GBLUP) which allows for joint evaluation of genotyped and non-genotyped animals [1]; see Legarra et al. [2] for a comprehensive review. The most widely used implementation involves a ‘simple’ extension of the pre-genomic ‘breeding value’ model, which replaces the pedigree-based numerator relationship matrix (NRM) between animals by its counterpart, which combines the genomic relationship matrix (GRM) between genotyped animals with relationships derived from the pedigree. Henceforth, a predicted breeding value that is obtained by using the GRM and SS-GBLUP will be referred to as GEBV while PEBV is used to denote the corresponding value based on the NRM ignoring genotype information, and EBV alludes to both types.

A problem inherent to combining genomically-derived and pedigree-based relationships arises due to different conceptual founder populations with potentially different means. For the NRM, the (unknown) parents of the first generation of pedigreed animals are considered to be the unrelated and non-inbred founder animals. Thus, the base generation is determined by the point in time where pedigree recording began. In contrast, genomic relationships are based on ancestral founders many generations back. Combining the NRM and GRM without accounting for these differences can result in biased predictions of breeding values, in particular EBV for genotyped animals may be biased downwards. This is akin to problems that have been encountered earlier on in genetic evaluation of beef cattle when PEBV of imported, superior bulls without appropriate local pedigree ties were found to be severely underestimated because they referenced the wrong, lower base [3]. Several procedures have been suggested to align the NRM and GRM [4–7]. Typically, such modifications have been found to reduce the overdispersion that is often reported for GEBV. However, observed effects on corresponding accuracies are generally small, e.g. [5, 8].

A standard measure, which is routinely computed to demonstrate the efficacy of selection programmes in livestock is the genetic trend. This is usually obtained as the mean EBV of cohorts of animals in a generation or born within a given time period. To date and to our knowledge, there are no studies that have examined estimates of genetic trend in the context of SS-GBLUP. This paper considers the effects of different modifications, which are suggested for the relationship matrices involved in SS-GBLUP, on the estimates of genetic trend. After a review of the methods proposed in the literature, we demonstrate by using simulated data that different ways of centering marker counts or aligning GRM and NRM can substantially affect estimates of trends, especially for populations that are subject to intense selection. This is followed by an application to data from LAMBPLAN, the Australian sheep genetic improvement scheme [9], representing a typical data structure where only relatively few animals have been genotyped so far and where these animals were born in the most recent years.

## Review: on the use of relationship matrices in SS-GBLUP

Consider a SS-GBLUP analysis comprised of $n_1$ non-genotyped and $n_2$ genotyped animals, with allele counts for *m* markers summarized in matrix **M** of size $n_2 \times m$. Assume standard coding of allele counts as 0 and 2 for homozygotes and 1 for heterozygotes. Let **A** and **G** denote the NRM and GRM, respectively, and **H** the joint relationship matrix. Assume animals are ordered so that **A** can be partitioned into blocks pertaining to genotyped (${\mathbf{A}}_{22}$) and non-genotyped animals (${\mathbf{A}}_{11}$) and the relationships between them (${\mathbf{A}}_{12}$). The joint relationship matrix is then [10]

1 $$\begin{aligned} {\mathbf{H}}&= \begin{pmatrix} {\mathbf{A}}_{11} + {\mathbf{A}}_{12} {\mathbf{A}}_{22}^{-1}\left( {\mathbf{G}}- {\mathbf{A}}_{22}\right) {\mathbf{A}}_{22}^{-1}{\mathbf{A}}_{21}&{}\quad {\mathbf{A}}_{12} {\mathbf{A}}_{22}^{-1}{\mathbf{G}}\\ {\mathbf{G}}{\mathbf{A}}_{22}^{-1}{\mathbf{A}}_{21}&{}\quad {\mathbf{G}}\end{pmatrix} \end{aligned}$$

with the inverse [11]

2 $$\begin{aligned} {\mathbf{H}}^{-1}&= {\mathbf{A}}^{-1}+ \begin{pmatrix} {\mathbf{0}} &{}\quad {\mathbf{0}} \\ {\mathbf{0}} &{}\quad {\mathbf{G}}^{-1}- {\mathbf{A}}_{22}^{-1}\end{pmatrix}. \end{aligned}$$

The genomic relationship is commonly determined as a matrix of sums of squares and crossproducts of the matrix of centered marker counts, possibly with some differential weighting for individual markers. A popular form is Van Raden’s [4] method 1:

3 $$\begin{aligned} {\mathbf{G}}_M&= \left( {\mathbf{M}}- 2 {\mathbf{P}} \right) \left( {\mathbf{M}}-2 {\mathbf{P}} \right) '/s \qquad {\text {with}} \qquad s= 2\sum _{i=1}^m p_i (1-p_i), \end{aligned}$$

where $p_i$ is the allele frequency of the *i*-th marker and **P** is a matrix comprised of columns $p_i{\mathbf{1}}$ with **1** a vector of length $n_2$ with all elements equal to unity. Subscript ‘M’ is used to denote the ‘raw’ GRM as derived from marker information, without any modifications. Other formulations, extend Eq. (3) to weigh contributions from individual markers according to their frequencies (e.g. method 2 of [4] or [12]).

Van Raden [4] emphasized that the frequencies $p_i$ should be those in the unselected base *a.k.a.* founder population. In practice, these are generally unknown and frequencies are commonly determined from the observed genotypes. Another choice is to assume that $p_i =0.5$ for all *i*, equivalent to coding allele counts as $-1,0$ and 1. An argument for the latter is that, for random choice of reference alleles, the expectation of $p_i$ is 0.5 [13]. Moreover, this coding is obtained when integrating the likelihood function for the single-step model over the unknown allele frequencies [6].

${\mathbf{G}}_M$ is often modified in some fashion to ensure that it can ‘safely’ be inverted, to improve alignment between the GRM and NRM or to account for residual polygenic variation. We use ${\mathbf{G}}^{\star }$ to denote the modified matrix with ${\mathbf{G}}$ on the right hand side of the following equations representing the matrix to be changed. Since different procedures can be combined, the latter may represent either ${\mathbf{G}}_M$ as given above or ${\mathbf{G}}^{\star }$ from a previous step.

### Weighted average of GRM and NRM

A common modification is to ‘shrink’ the GRM towards the corresponding part of the NRM:

4 $$\begin{aligned} {\mathbf{G}}^{\star }&= \lambda \, {\mathbf{G}}+ \left( 1 - \lambda \right) {\mathbf{A}}_{22}\qquad {\text {with}} \qquad 0 \le \lambda \le 1 \end{aligned}.$$

Often values of $\lambda$ close to unity [4] are used to counter-act the problem of ${\mathbf{G}}_M$ not being positive definite when observed allele frequencies are used to center **M**. Smaller values of $\lambda$ are used for analyses where it is deemed necessary to account for residual polygenic variation, i.e. additive genetic variance not explained by the markers, or to limit the influence of genomic information. For instance, values of $\lambda = 0.5$ have been chosen for SS-GBLUP genetic evaluation of Australian sheep [14] and beef cattle [15].

### Adjusting the GRM to be compatible with the NRM

Suggestions for aligning ${\mathbf{G}}$ with ${\mathbf{A}}$ or ${\mathbf{A}}_{22}$ involve a modification of the form:

5 $$\begin{aligned} {\mathbf{G}}^{\star }&= \beta {\mathbf{G}} + \alpha {\mathbf{J}} {\text {,}} \end{aligned}$$

where ${\mathbf{J}}$ denotes a matrix with all elements equal to unity. Factors $\alpha$ and $\beta$ can be estimated by least-squares regression [4] or determined by equating the means of the elements of the two matrices, **G** and ${\mathbf{A}}_{22}$, and the means of their diagonals [6]. The latter may seem heuristic, but can be thought of as enforcing equality of a sample covariance matrix and its expectation for both matrices [7]. Legarra et al. [2] interpreted $\alpha$ as ‘overall relationship’ and $\beta$ as change in scale or genetic variance due to drift or selection.

This gives:

6 $$\begin{aligned} \alpha&= \left[ {{\mathrm{tr}}}\left( {\mathbf{A}}_{22}\right) - {{\mathrm{tr}}}\left( {\mathbf{G}}\right) \right] / n_2\end{aligned}$$

7 $$\begin{aligned} \beta&= \left[ {{\mathrm{tr}}}\left( {\mathbf{A}}_{22}\right) - {\mathbf{1}}' {\mathbf{A}}_{22}{\mathbf{1}} \Big / n_2 \right] \Big / \left[ {{\mathrm{tr}}}\left( {\mathbf{G}}\right) - {\mathbf{1}}' {\mathbf{G}}{\mathbf{1}} \Big / n_2 \right] {\text {,}} \end{aligned}$$

where **1** is a vector with all elements equal to unity and ‘${{\mathrm{tr}}}$’ denotes the matrix trace operator.

Similarly, Vitezica et al. [5] proposed values of

8 $$\begin{aligned} \alpha = \left( {\mathbf{1}}' {\mathbf{A}}_{22}{\mathbf{1}} - {\mathbf{1}}' {\mathbf{G}}{\mathbf{1}} \right) \big / n_2^2 \qquad {\text {and}} \qquad \beta = 1 \end{aligned}.$$

This yields a value of $\alpha$ which is equal to the mean difference between ${\mathbf{A}}_{22}$ and **G**, so that the means of elements of the modified GRM and the corresponding part of the NRM are equal.

Several studies recognized that adding a multiple of **J** to the GRM shifts GEBV for genotyped animals by a constant, i.e. is inconsequential for analyses which do not include individuals without genotypes. For instance, Stranden and Christensen [16] showed that allele coding did not affect relative differences between predicted genomic breeding values, provided the model included a fixed mean effect. Comparing different additions to the GRM, Tier et al. [17] demonstrated that adding very different multiples of **J** yielded analogous results.

Vitezica et al. [5] emphasized that replacing **G** by ${\mathbf{G}} + \alpha {\mathbf{J}}$ implies fitting a mean term $\mu$ assumed to have variance $\alpha \sigma _g^2$ (with $\sigma _g^2$ the additive genetic variance), arguing that the mean of random breeding values (of genotyped animals) should also be a random effect. The authors further showed that this is an equivalent model to fitting a single genetic group for genotyped animals explicitly with group proportion for their non-genotyped relatives given by ‘pedigree regression’, ${\mathbf{A}}_{12} {\mathbf{A}}_{22}^{-1}{\mathbf{1}}$. Similarly, Fernando et al. [18] proposed to fit a (fixed) mean for genotyped animals in SS-GBLUP implementations fitting a marker effect or hybrid model (rather than the breeding value model) to account for inappropriate centering of allele counts or imputation error. A simulation study considering such models for populations under selection, confirmed that estimates for $\mu$ represented the mean GEBV of genotyped individuals when observed genotypes were centered by their mean frequencies [19].

Moreover, Vitezica et al. [5] suggested that $\alpha$ could be interpreted as twice the so-called ${F}_{\hbox {ST}}$ or fixation index, which gives the average relationships of gametes for a given base population. They pointed out that the ${F}_{\hbox {ST}}$ based adjustment to change base population described by Powell et al. [20] translates to a modification of ${\mathbf{G}}$ with $\alpha$ as above [see Eq. (5)] and $\beta = \left( 1 - \alpha /2 \right)$.

However, please note that, depending on the choice of values for $\alpha$ and $\beta$, ${\mathbf{G}}^{\star }$ is not guaranteed to be positive definite. Interpretation of $\alpha$ as a variance implies a positive value. The adjustments of form of Eq. (5) were proposed for the scenario in which markers were centered using their observed frequencies—different choices for **P** could readily yield elements of ${\mathbf{G}}_M$ much larger than of ${\mathbf{A}}_{22}$ and thus a negative estimate for $\alpha$ or an invalid ${\mathbf{G}}^{\star }$, and should not be used naively.

### Modifying the NRM to match the GRM

An alternative is to scale the NRM to be similar to the GRM, so as to account for ancestral relationships that are captured by genomic information but not the pedigree. This is similar to earlier attempts to account for prior inbreeding in genetic evaluation; see Van Raden [21]. Let $\gamma$ represent the degree of ‘self-relationship’ among the base animals in the pedigree. Christensen [6] then proposed to replace **A** with:

9 $$\begin{aligned} {\mathbf{A}}_\gamma&= \left( 1 - \gamma /2 \right) {\mathbf{A}} + \gamma {\mathbf{J}} {\text {.}} \end{aligned}$$

Using the Sherman–Morrison matrix identity, gives the inverse:

10 $$\begin{aligned} {\mathbf{A}}^{-\gamma }&= \frac{2}{ 1 - \gamma } \left[ {\mathbf{A}}^{-1}- \frac{\gamma }{1 - \gamma /2 + \gamma F } \begin{pmatrix} {\mathbf{J}}_{F} &{} {\mathbf{0}} \\ {\mathbf{0}} &{} {\mathbf{0}} \end{pmatrix} \right] \end{aligned}$$

as ${\mathbf{1}}' {\mathbf{A}}^{-1}= \bigl ( {\mathbf{1}}_{F}' \vdots \, {\mathbf{0}} \bigr )$, with *F* denoting the number of founders in the pedigree.

This modification is of the same form as the ${F}_{\hbox {ST}}$ based adjustment of **G** [20]. Indeed, Garcia-Baccino et al. [13] showed that $\gamma$ can also be interpreted as twice the ${F}_{\hbox {ST}}$ index. Hence, it attempts to change the base population for pedigreed individuals.

Legarra et al. [7] subsequently demonstrated that the same adjustment can be obtained by augmenting the pedigree with a so-called meta-founder, a conceptual parent which replaces the unknown parents of founder animals in the pedigree, acting as both sire and dam. This framework is attractive as it allows for computation of the terms required to build ${\mathbf{H}}^{-1}$, specifically ${\mathbf{A}}^{-\gamma }$ and the submatrix of ${\mathbf{A}}_\gamma$ for genotyped animals, ${\mathbf{A}}_{22}^{\gamma }$, with minor modifications of commonly used existing algorithms for these tasks. Moreover, multiple base populations are readily accommodated by allowing for separate metafounders and replacing the scalar $\gamma$ with a positive definite matrix ${\varvec{\Gamma }}$ with diagonal elements equal to the individual self-relationships and off-diagonal elements comprised of across population relationships. This makes it suitable for the analysis of crossbred populations; see, for instance, [22] for an application. Alternatively, metafounders can be thought of as a generalisation of the ‘phantom parents’ to model genetic groups for unknown parents for genetic evaluation using pedigree information [23].

Let ${\mathbf{A}}^{-{\varvec{\Gamma }}}$ and ${\mathbf{A}}_{22}^{{\varvec{\Gamma }}}$ denote the equivalents to ${\mathbf{A}}^{-\gamma }$ and ${\mathbf{A}}_{22}^{\gamma }$ for multiple meta-founders. Both [6] and [7] presented algorithms to evaluate the terms required to set up ${\mathbf{H}}^{-1}$, ${\mathbf{A}}^{-\gamma }$ or ${\mathbf{A}}^{-{\varvec{\Gamma }}}$ and ${\mathbf{A}}_{22}^{\gamma }$ or ${\mathbf{A}}_{22}^{{\varvec{\Gamma }}}$ recursively, extending the procedures of Quaas [24] and Colleau [25]. Similarly, both described likelihood based approaches to estimate $\gamma$ or ${\varvec{\Gamma }}$. Centering marker counts by $2{\mathbf{P}} = {\mathbf{J}}$ and with ${\mathbf{M}}_C= {\mathbf{M}} - {\mathbf{J}}$ denoting the centered matrix of allele counts, this requires maximising:

11 $$\begin{aligned} \log L&\propto -\frac{m}{2} \left( n_2 \log ( s ) + \log \left| {\mathbf{A}}_{22}^{{\varvec{\Gamma }}} \right| + \frac{1}{s} {{\mathrm{tr}}}\left( {\mathbf{A}}_{22}^{-{\varvec{\Gamma }}} {\mathbf{M}}_C{\mathbf{M}}_C'\right) \right) \end{aligned}$$

with respect to the elements of ${\varvec{\Gamma }}$, with

12 $$\begin{aligned} s&= {{\mathrm{tr}}}\left( {\mathbf{A}}_{22}^{-{\varvec{\Gamma }}} {\mathbf{M}}_C{\mathbf{M}}_C'\right) \Big / n_2 \end{aligned}.$$

Variable *s* represents a measure of heterozygosity, similar to $s = 2 \sum _i p_i (1-p_i)$ above. While estimation of ${\varvec{\Gamma }}$ from Eq. (11) involves numerical optimisation, Eq. (12) is of closed form and *s* can be obtained directly. Alternatively, ${\varvec{\Gamma }}$ and *s* can be estimated based on summary statistics. For a single metafounder [7],

13 $$\begin{aligned} s&= \frac{ n_2 {{\mathrm{tr}}}\left( {\mathbf{M}}_C{\mathbf{M}}_C'\right) \bigl [ 1 - {\mathbf{1}}' {\mathbf{A}}_{22} {\mathbf{1}} / (2n_2^2) \bigr ] - {\mathbf{1}}' {\mathbf{M}}_C{\mathbf{M}}_C'{\mathbf{1}} \bigl [ 1 - {{\mathrm{tr}}}\left( \mathbf{A}_{22} \right) /(2 n_2) \bigr ] }{ n_2 {{\mathrm{tr}}}( {\mathbf{A}}_{22}) - {\mathbf{1}}' {\mathbf{A}}_{22}{\mathbf{1}}} \end{aligned}$$

and

14 $$\begin{aligned} \gamma&= \frac{{\mathbf{1}}' {\mathbf{M}}_C{\mathbf{M}}_C'{\mathbf{1}} / s - {\mathbf{1}}' {\mathbf{A}}_{22}{\mathbf{1}}}{ n_2^2 - {\mathbf{1}}' {\mathbf{A}}_{22}{\mathbf{1}}/2} \end{aligned}.$$

Subsequently, Garcia-Baccino et al. [13] described generalised least squares estimators for $\gamma$ or ${\varvec{\Gamma }}$. For a single metafounder,

15 $$\begin{aligned} \hat{\mu }_i = \left( {\mathbf{1}}' {\mathbf{A}}_{22}^{-1}{\mathbf{1}} \right) ^{-1} {\mathbf{1}}' {\mathbf{A}}_{22}^{-1}{\mathbf{m}}_i \end{aligned},$$

where ${\mathbf{m}}_i$ denotes the vector of *un*centered allele counts for the *i*-th locus ($i=1,m$). An estimate of $\gamma$ is then obtained as twice the variance of $\hat{\mu }_i$ across loci. Furthermore, [13] outlined corresponding maximum likelihood schemes, based on the assumption that the $\mu _i$ are normally and independently distributed. The authors presented a simulation study for a single metafounder, reporting that both methods estimated $\gamma$ accurately while the summary statistics based approach tended to yield overestimates.

As noted by Strandén et al. [26], quantities $\hat{\mu }_i$ given in Eq. (15) are estimates of (twice) the founder allele frequencies as proposed by McPeek et al. [27]. These are readily calculated for large numbers of genotyped animals, using:

16 $$\begin{aligned} {\mathbf{A}}_{22}^{-1}&= {\mathbf{A}}^{22} - {\mathbf{A}}^{21} \left( {\mathbf{A}}^{11}\right) ^{-1} {\mathbf{A}}^{12} \end{aligned},$$

where ${\mathbf{A}}^{ij}$ are the submatrices of ${\mathbf{A}}^{-1}$ corresponding to the partitioning of ${\mathbf{A}}$ for genotyped and non-genotyped individuals. Hence, ${\mathbf{A}}_{22}^{-1}{\mathbf{1}}$ in Eq. (15) can be obtained by using sparse matrix calculations without the need to invert a large matrix, requiring the factorisation of ${\mathbf{A}}^{11}$ instead [26].

### ‘Correction factors’ in building ${\mathbf{H}}^{-1}$

In addition, it has been suggested to weigh ${\mathbf{G}}^{-1}$ and ${\mathbf{A}}_{22}^{-1}$  differently when constructing ${\mathbf{H}}^{-1}$, i.e.

17 $$\begin{aligned} {\mathbf{H}}^{-1}&= {\mathbf{A}}^{-1}+ \begin{pmatrix} {\mathbf{0}} &{}\quad {\mathbf{0}} \\ {\mathbf{0}} &{}\quad \tau {\mathbf{G}}^{-1}- \omega {\mathbf{A}}_{22}^{-1}\end{pmatrix} \end{aligned}$$

or similar. Suitable values for $\tau$ and $\omega$ were commonly determined experimentally by evaluating their effect on the inflation of genomic breeding values. Reduction in bias for values different from unity with little effect on accuracy have been reported for dairy cattle genetic evaluation [28, 29]. In particular, reducing the weight on ${\mathbf{A}}_{22}^{-1}$ appeared advantageous ($\omega < 1$) by reducing the effects of a high proportion of incomplete pedigrees [30]. Garcia-Baccino et al. [13] emphasized that the metafounder approach would act in a similar fashion, albeit with a theoretically justified basis. Martini et al. [31] showed that weighting of ${\mathbf{G}}^{-1}$ and ${\mathbf{A}}_{22}^{-1}$ as in Eq. (17) is equivalent to replacing the diagonal block for genotyped animals in **H** [see Eq. (1) above] with:

18 $$\begin{aligned} {\mathbf{G}}^{\star }&= \left( \tau {\mathbf{G}}^{-1}+ (1-\omega ) {\mathbf{A}}_{22}^{-1}\right) ^{-1} \end{aligned},$$

which can be thought of as a weighted harmonic mean of ${\mathbf{G}}$ and ${\mathbf{A}}_{22}$. Note that, depending on the choice of $\tau$ and $\omega$, ${\mathbf{G}}^{\star }$ again is not guaranteed to be positive definite.

## Methods

### Simulation

To examine the effects of different methods of aligning ${\mathbf{G}}$ and ${\mathbf{A}}$, data were simulated using the software package QMSim  [32] adopting the scenario used by [5] and [13] to mimic a livestock population under selection (Parameter file available at http://github.com/alegarra/metafounder); see their papers for details. This was modified slightly to consider a trait recorded on both sexes and by reducing the number of markers. In brief, this yielded records and genotypes comprised of allele counts for 46,500 loci for 2800 unselected animals with unknown parents in generation 0 and 2600 animals in each of 10 overlapping generations, 28,800 in total. Records were sampled for a trait with a phenotypic variance of 10 and heritability of 0.3. Parents of the next generation were selected based on their breeding values, obtained by BLUP using the (pedigree) numerator relationship matrix. Selection involved replacement of 80 out of 200 sires and 520 out of 2600 dams in each generation. A total of 25 replicates were obtained and analysed.

Data were analysed by considering successive subsets of genotypes in generations *i* through to 10, for $i = 0, 2, 4, 7$ and 10. Markers with minor allele frequencies lower than 0.02 were disregarded. In addition, a ‘pedigree BLUP’ analysis was carried out, ignoring all genotypes. Records and pedigree information for all generations were used throughout. Restricted maximum likelihood analyses to estimate genetic and residual variances were carried out by fitting a linear model with an overall mean as the only fixed effect and animals’ genetic merit as random effects, obtaining predicted breeding values at convergence.

The inverse of the joint relationship matrix between genotyped and non-genotyped animals was built in three ways. First, ${\mathbf{H}}^{-1}$ was constructed without any attempt to align ${\mathbf{G}}$ and ${\mathbf{A}}$, referred to as model M0. Second, for model MG the GRM was augmented by $\alpha {\mathbf{J}}$ as proposed by Vitezica et al. [5]. Preliminary analyses had shown negligible differences in results for MG to corresponding analyses using the forms of adjustment suggested by [6] or [20], and the latter were thus not examined any further. Lastly for model MA, ${\mathbf{A}}^{-1}$ and ${\mathbf{A}}_{22}^{-1}$ in ${\mathbf{H}}^{-1}$ [see Eq. (2)] were replaced with ${\mathbf{A}}^{-\gamma }$ and its submatrix for genotyped individuals, ${\mathbf{A}}_{22}^{-\gamma }$, with ${\mathbf{G}}^{-1}$ as for M0. This was done by augmenting the mixed model with a single meta-founder. The degree of self-relationship, $\gamma$, for each subset of genotypes considered was estimated using the generalised least-squares procedure described by Garcia-Baccino et al. [13] and ${\mathbf{A}}^{-\gamma }$ and ${\mathbf{A}}_{22}^{-\gamma }$ were built following Legarra et al. [7].

In turn, ${\mathbf{G}}_M$ was constructed using up to four different sets of allele frequencies to center **M**. The first, denoted as all, was calculated using all observed genotypes in the subset, as is common practice. Second, only the genotypes for the first generation available were used, yielding case 1st. For comparison, the third scheme, fou, considered the founder frequencies, i.e. for individuals in generation 0. Finally, all frequencies were assumed to be equal to 0.5 (half). No weighted averaging between ${\mathbf{G}}$ and ${\mathbf{A}}$ was performed, i.e. $\lambda =1$ was used. Instead, safely positive definite matrices ${\mathbf{G}}$ were ensured by augmenting their diagonal elements with a constant of 0.05.

The examined summary statistics included mean EBV for each generation and coefficients for the regression of true on predicted breeding values for individuals in generation 10. In addition, mean GEBV per generation for each genomic analysis were deviated from the corresponding PEBV values, after standardising the ‘location’ of curves by subtracting the mean GEBV or PEBV for generation 0 from the respective means for all generations. A measure of discrepancy between estimates of genetic trends for different analyses was then calculated as the Frobenius norm of the resulting vector. Standardisation ensured that this quantity reflected only differences in shape of the trajectories.

Analyses were carried out using our mixed model software WOMBAT, building ${\mathbf{H}}^{-1}$ by using a recently added module to carry out the associated calculations [33, 34].

### Application

Methods were also tested for Australian sheep data, using records from the LAMBPLAN [9] maternal breeds genetic evaluation for the trait ‘number of lambs born in one year old ewes’ (ynlb), defined as number of lambs born per ewe mated. A total of 19,564 ynlb records were collected on ewes born between 2007 and 2016. Of the ewes with records, 905 were genotyped. In addition, there were 275 animals in the pedigree with genotypes available but no records, mostly sires. This yielded a total of 1180 genotypes included in the SS-GBLUP analyses. Genotypes were comprised of marker allele counts from either 12 or 50 K ovine SNP chips (Illumina Inc., San Diego, CA, USA), with 12 K genotypes imputed to 50 K. Table 1 summarizes numbers of records and numbers of genotyped ewes per year of birth, showing that genotyped animals were concentrated in the more recent data. The pedigree records available included 34,947 animals, extending back to animals born in the late 1980’s. However only animals born from the year 2000 onwards were considered in the calculation of genetic trends.

**Table 1:** Distribution of numbers of records and ewes with genotypes and phenotypes across years of birth

Year	2007	2008	2009	2010	2011	2012	2013	2014	2015	2016
No. records	953	835	1273	1000	1819	1535	2490	2674	3829	3156
No. genotypes	–	–	–	–	89	147	37	118	514	–

Data for ynlb were analysed using WOMBAT which involved fitting single trait animal models with a fixed effect for contemporary group (250 levels) and an additional random effect for service sire (291 levels). The genetic effect was again fitted either without genotypes as ‘pedigree BLUP’, or with genotypes as SS-GBLUP fitting ${\mathbf{H}}^{-1}$ as described above: for M0, no attempt was made to align ${\mathbf{G}}$ with ${\mathbf{A}}$, for MG, ${\mathbf{G}}$ was augmented with $\alpha {\mathbf{J}}$, and for MA, the meta-founder approach with $\gamma$ estimated from the data was used. To construct ${\mathbf{G}}_M$, all observed genotypes (all) were used to calculate allele frequencies. In addition, analyses were repeated assuming frequencies of 0.5 (half) for MA only. For M0, two values of the parameter $\lambda$ (see Eq. (4)) were used to compute a weighted average of ${\mathbf{G}}$ and ${\mathbf{A}}$, 1 as above and 0.5 as used in routine SS-GBLUP evaluations for Australian sheep [14].

## Results

### Simulation

**Fig. 1:**
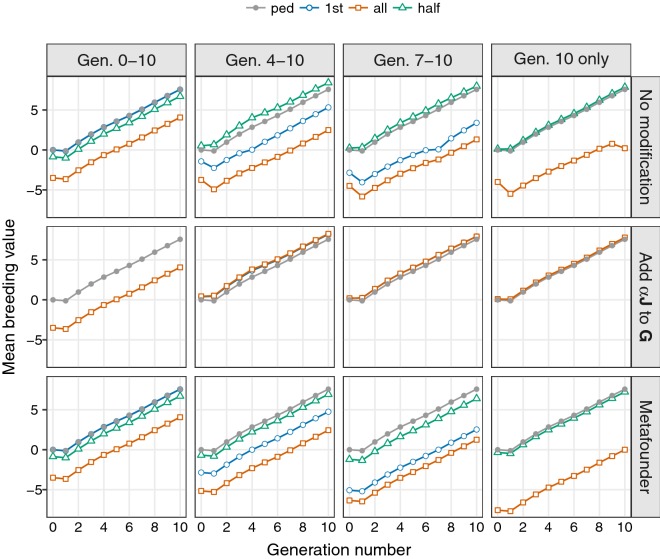
Estimates of genetic trend^[a]^ for a single replicate for different scaling methods and allele frequencies^[b][c]^. ^[a]^Simulated data, ^[b]^Allele frequencies: ped ignoring genotypes (pedigree analysis), 1st using observed frequencies in first generation available, all using observed frequencies from all genotypes, and half assuming frequencies of 0.5, ^[c]^Considering genotypes in selected generations: 0 to 10, 4 to 10, 7 to 10 and 10 only

Estimates of genetic trend for different analyses and amounts of genotypes available are shown in Fig. 1 for a single replicate (results for the subset comprising generations 2 to 10 were omitted). This is representative of the typical pattern observed for all replicates. All panels show mean PEBV as a reference. The latter were virtually identical to corresponding true means, scaled to zero for generation 0 (not shown). Considering genotypes for generation 10 only, allele centering strategies all and 1st are the same and only all is shown. For model MG and 1st, the estimate of $\alpha$ when considering all genotypes was negative and ${\mathbf{G}}^{\star }$ was not positive definite, causing the analysis to fail.

Without modifications of ${\mathbf{A}}$ or ${\mathbf{G}}$, using observed frequencies (all) to center allele counts, mean GEBV were shifted downwards. The resulting trend curve was parallel to the corresponding curve of pedigree means when all animals were genotyped, i.e. individuals were ranked correctly and, without the need to align GEBV for genotyped and non-genotyped animals, the shift is inconsequential. Indeed, in practical evaluation schemes, estimates are often scaled to a selected, fixed base, i.e. the ‘shape’ rather than ‘location’ of estimated trend trajectories is important.

As fewer generations of genotypes were considered, the discrepancy between curves increased. For this method of centering, the mean of the genotyped animals used to determine the allele frequencies is forced to be zero [4]. As especially evident when considering genotypes in generation 10 only, this can lead to a marked distortion of the estimated trend. Conversely, as expected, using founder frequencies (generation 0, fou) resulted in trend estimates that are indistinguishable from the pedigree values for all subsets of genotypes (not shown). Consequently, using only the first generation of observed genotypes (1st) to estimate allele frequencies resulted in less biased estimates of trends but again altered the shape of the trajectory by forcing the mean GEBV for that generation to be zero. Similarly, assuming gene frequencies of 0.5 yielded mean GEBV per generation that are reasonably close to the pedigree values. In part, this may be attributable to the fact that the mean allele frequency (across loci and replicates) in generation 0 was 0.48, i.e close to 0.5. However, whereas the average proportion of loci with frequencies in the middle deciles (0.4–0.6) was 24%, 13% of markers had frequencies in the extreme deciles ($<0.1$ or $\ge 0.9$).

Estimates of $\alpha$ for MG for all increased with the first generation number for which genotypes were considered: values were 0.027, 0.041, 0.071 and 0.111 for genotypes in generations 2 to 10, 4 to 10, 7 to 10 and 10 only, respectively (if all genotypes were considered, the estimate was close to zero, 0.018, i.e. there was virtually no modification, as for M0). This yielded mean GEBV very close to mean PEBV when only genotypes in the last or last few generations were used, but less close agreement otherwise. The simulation involved strong selection and associated sizeable changes in allele frequencies over generations. While $\alpha$ corrects for changes in mean due to selection or drift, it does not allow for the accompanying reduction in genetic variance from the conceptual base population [2]. Results suggest that estimates of a global $\alpha$ for all generations may not be sufficient if many generations are genotyped. Centering using fou or half for MG was not considered as these resulted in negative estimates of $\alpha$ and thus non-positive definite matrices ${\mathbf{G}}^{\star }$.

Finally, modifying ${\mathbf{A}}$ to align with ${\mathbf{G}}$ yielded parallel curves for all four types of centering. Estimates of $\gamma$ decreased slightly as fewer generations of genotypes were considered, 0.548, 0.543, 0.540, 0.539 and 0.537 for generations 0 to 10 to generation 10 only. As above, using all observed genotypes (all) to construct ${\mathbf{G}}$ resulted in the largest shift, with the mean GEBV for genotyped animals forced to zero. However, fitting a metafounder yielded an estimate of the shift. For instance, for all these were − 3.50 (0–10), − 4.34 (2–10), − 5.16 (4–10), − 6.34 (7–10) and − 7.56 (10 only). Subtracting these estimates from the corresponding mean GEBV then shifted the trajectories to be superimposed on the curve for mean PEBV. This held for all types of frequencies. Assuming frequencies of 0.5, GEBV for the metafounder were expected to be zero, i.e. GEBV should have been aligned correctly. In practice, there were small deviations. This may, at least in part, be attributed to sampling or other errors in the estimate of $\gamma$. Christensen [6] emphasized that, strictly speaking, $\gamma$ should be estimated using observed phenotypes as well as genotypes, and reported slight overestimates when ignoring phenotypes.

**Table 2:** Selected means ($\bar{x}$) and standard deviations (SD) across replicates for the norm of the vector of mean breeding values per generation deviated from ‘pedigree only’ values and for the regression coefficients of true on predicted breeding value for animals in generation 10

M. $^{\mathrm{{a}}}$	Fr. $^{\mathrm{{b}}}$	Genotypes in generations									
		0–10		2–10		4–10		7–10		10 Only	
		$\bar{x}$	SD	$\bar{x}$	SD	$\bar{x}$	SD	$\bar{x}$	SD	$\bar{x}$	SD
*Norm for vector of mean breeding values*											
M0	all	0.10	0.04	1.19	0.03	1.39	0.07	1.68	0.12	2.53	0.09
M0	1st	0.10	0.04	0.41	0.03	1.27	0.08	1.82	0.13	2.53	0.09
M0	fou	0.10	0.04	0.07	0.01	0.06	0.02	0.05	0.02	0.04	0.01
M0	half	0.09	0.04	0.60	0.02	0.65	0.04	0.44	0.05	0.13	0.01
MG	all	0.10	0.04	0.41	0.04	0.47	0.05	0.32	0.03	0.09	0.01
MA	half	0.09	0.04	0.09	0.02	0.09	0.02	0.06	0.02	0.01	0.01
*Regression of true on predicted breeding values*											
M0	all	0.992	0.020	0.883	0.021	0.841	0.028	0.784	0.033	0.728	0.035
M0	1st	0.992	0.020	0.979	0.020	0.921	0.020	0.806	0.026	0.728	0.035
M0	fou	0.992	0.020	0.991	0.021	0.989	0.021	0.980	0.021	0.944	0.023
M0	half	0.989	0.020	1.031	0.023	1.058	0.026	1.114	0.038	1.274	0.055
MG	all	0.992	0.020	0.998	0.023	1.017	0.025	1.049	0.031	1.109	0.041
MA	half	0.989	0.020	0.990	0.021	0.993	0.021	0.999	0.023	1.001	0.027

$^{\mathrm{{a}}}$ Model M0: No alignment between GRM and NRM, MG: Modifying the GRM by adding $\alpha {\mathbf{J}}$, and MA: Extending the NRM to include a meta-founder with self-relationship $\gamma$

$^{\mathrm{{b}}}$ Frequencies used to center marker allele counts—all: using all genotypes in the subset, 1st: using only genotypes in the first generation available, fou: using founder frequencies (generation 0), and half: assuming frequencies of value 0.5 throughout

There was little variation in results over replicates. Table 2 summarises selected means of the Frobenius norm of the vector of differences between mean EBV from pedigree and genomic analyses and their standard deviations across replicates. Mean Frobenius norms confirm the observations on a single replicate above: if founder allele frequencies were known and used to construct ${\mathbf{G}}$ no alignments would be needed. In the absence thereof, fitting a metafounder and using the resulting estimates of its effect to account for the shift in GEBV due to selection yields comparable results. We also provide the corresponding statistics for the regression of true on predicted breeding values for individuals in the last generation. Mean regression coefficients were close to the theoretical value of unity when all animals were genotyped, using founder frequencies to center allele counts or fitting a metafounder. For M0 with frequencies half and MG with all, some under-dispersion of GEBV (regression coefficients $>1$) was apparent when only individuals in the last few generations were genotyped. This was not reflected in the corresponding mean norm values. For pedigree only analyses, the mean regression was 0.984 with standard deviation of 0.034.

Simulations considered the scenario where all animals in a generation were either genotyped or not. In practice, this is unlikely. Simulations were thus repeated deleting genotype information for every third animal (not shown). For case M0 using observed frequencies, either all or 1st, reduced regression coefficients further, the more so the fewer genotyped generations were considered. For instance, for all and genotypes in generation 10 only, the regression of true on predicted breeding values dropped to 0.55. Whereas standard deviations across replicates increased slightly, regression coefficients for the other cases differed little from the values given in Table 2, i.e. they remained close to unity. Corresponding mean Frobenius norms (not shown) were somewhat smaller as fewer individuals were genotyped.

### Application

**Fig. 2:**
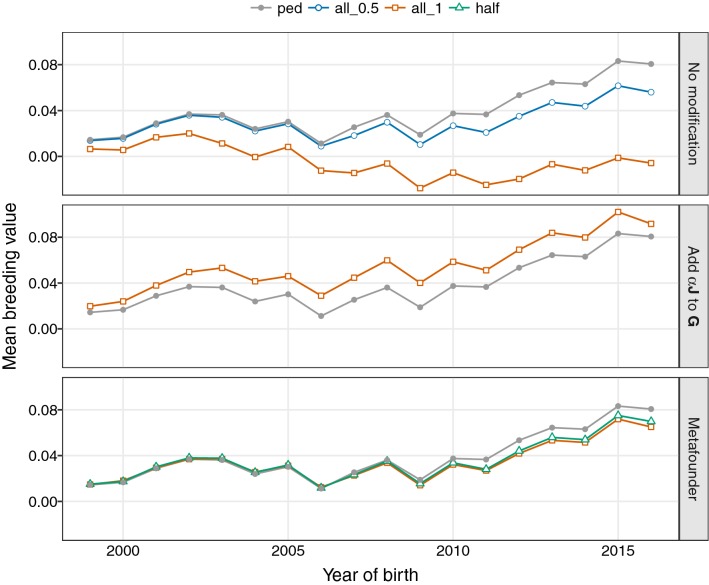
Estimates of genetic trend^[a]^ for different methods of scaling relationship matrices and assumed allele frequencies^[b]^. ^[a]^Sheep data for trait ‘number of lambs born in one year old ewes’, ^[b]^Allele frequencies: ped ignoring genotypes (pedigree analysis) all_0.5 using observed frequencies (all genotypes) for $\lambda = 0.5$, all_1 using observed frequencies (all genotypes) for $\lambda = 1$, and half assuming frequencies of 0.5

Estimates of genetic trend for different analyses of the sheep data are shown in Fig. 2. Corresponding values of the Frobenius norm for deviations from ‘pedigree BLUP’ results are summarized in Table 3, together with estimates of heritabilities and phenotypic variances.

**Table 3:** Estimated heritabilities and norm of the vector of mean breeding values per generation deviated from ‘pedigree only’ values for different SS-GBLUP analyses

Model$^{\mathrm{{a}}}$	*λ*	Freq.$^{\mathrm{{b}}}$	* h* ^2c^	$\sigma _P^{2}$ $^{\mathrm{{d}}}$	Norm
M0	1.0	all	0.08	0.56	0.185
	0.5	all	0.08	0.56	0.049
MG	1.0	all	0.09	0.56	0.049
MA	1.0	all	0.11	0.57	0.031
	1.0	half	0.09	0.58	0.025

$^{\mathrm{{a}}}$ M0: No alignment between GRM and NRM, MG: Modifying the GRM by adding $\alpha {\mathbf{J}}$, and MA: Extending the NRM to include a meta-founder with self-relationship $\gamma$

$^{\mathrm{{b}}}$ Frequencies to center marker allele counts – all: using all genotypes in the subset and half: assuming frequencies of value 0.5 throughout

$$^{\mathrm{{c}}}$$ Heritability

$$^{\mathrm{{d}}}$$ Phenotypic variance

As for the simulated data, estimates of genetic trend for SS-GBLUP without any attempts to align ${\mathbf{G}}$ and ${\mathbf{A}}$ (top panel) differed substantially from the pedigree-based estimates, especially for the years with genotyped animals. Reducing the influence of genomic information by replacing the GRM with the average of ${\mathbf{G}}$ and ${\mathbf{A}}_{22}$ ($\lambda = 0.5$) reduced differences markedly. Modifying ${\mathbf{G}}$ as suggested by Vitezica et al. [5] yielded a GEBV trajectory which was mostly parallel to that for pedigree only analyses (middle panel; considering $\lambda =1$ only), i.e. of the correct shape but with some shift in location evident (for an estimate of $\alpha =0.0263$).

Results for analyses fitting a meta-founder shown in Fig. 2 are mean GEBV adjusted for the estimate of the meta-founder effect (in contrast to corresponding results in Fig. 1 which are mean GEBV prior to adjustment for the meta-founder). The predicted value for the meta-founder was − 0.077 for $\lambda =1$ and an estimate of $\gamma = 0.48$. Hence, estimated trends for all analyses agreed well, although there was a tendency for mean GEBV in the last few years to be slightly lower than corresponding mean PEBV. Presumably this is, at least partially, a reflection of the small number of genotypes available and resulting sampling errors.

Estimates of heritability and phenotypic variances varied little between analyses, with the estimated variance ratio for the service sire effect equal to 0.04 throughout. Values for the discrepancy in genetic trends between SS-GBLUP and pedigree BLUP analyses reflected observations for Fig. 2, with small numerical values which are an artifact of low phenotypic variances.

## Discussion

Joint genetic evaluation of genotyped and non-genotyped animals in a single-step analysis has become a standard procedure. Results clearly illustrate that care has to be taken to model different means and conceptual founder populations appropriately, especially for populations under selection. In particular, estimates of genetic trends are easily distorted and can differ with the assumptions on gene frequencies or the way they are estimated. As reviewed briefly, various methods have been suggested to combine or align the pedigree-based and genomically-derived relationship matrices or scale selected components of ${\mathbf{H}}^{-1}$ to improve the accuracy or reduce the bias in GEBV from SS-GBLUP analyses. Moreover, most of these are easy to apply.

Current livestock improvement schemes typically have genotype information for the most recent generation(s) of animals only, especially for the more extensive industries such as sheep and beef cattle. Results show that both scaling of ${\mathbf{G}}$ to align with ${\mathbf{A}}$  or, vice versa, scaling ${\mathbf{A}}$ to match ${\mathbf{G}}$ are effective for this scenario, with estimates of genetic trends in good agreement with true values (simulation) or results from pedigree only analyses. However, when more generations of genotypes are considered, estimated trajectories tend to be shifted somewhat (although still of the correct shape), especially when centering marker allele counts using all observed genotypes to estimate gene frequencies. In this case, the meta-founder approach has an immediate advantage: the GEBV for the meta-founder provides an estimate for the shift in GEBV—and adjusting for it yields the correct location for the curve. The expectation for the latter is a mean of zero for the founder generation. For practical applications where models of analysis include many fixed effects (as in our sheep example) this may differ somewhat if fixed effects and animals are sufficiently confounded so that fixed effects remove some of the trend. Analogously, the shift may be estimated for MG by fitting an equivalent model including a genetic group effect [5], but this was not considered.

Moreover, for the meta-founder approach coefficients for the regression of true on predicted breeding values (simulation) were essentially equal to unity, while adding $\alpha {\mathbf{J}}$ to ${\mathbf{G}}$  resulted in slight deflation (coefficients $>1$) when only genotypes in recent generations were considered. Adjusting ${\mathbf{A}}$ to align with ${\mathbf{G}}$ makes the genomic base of the reference population. The parameter $\gamma$, estimated from the genomic information and ranging from 0 to 2, can be interpreted as the degree of homozygosity among the pedigree founders that would yield observed relationships closest to those in ${\mathbf{G}}$, where ${\mathbf{G}}$ is obtained assuming allele frequencies equal to 0.5. In other words, ${\mathbf{G}}$ refers to a conceptual, genomic base with maximum variability for all loci [35].

## Conclusions

Alignment of pedigree-based and genomic relationship matrices for single-step genetic evaluation of populations under selection is essential. Making the pedigree based relationship to be compatible with genomic information by fitting meta-founders is simple and effective.
